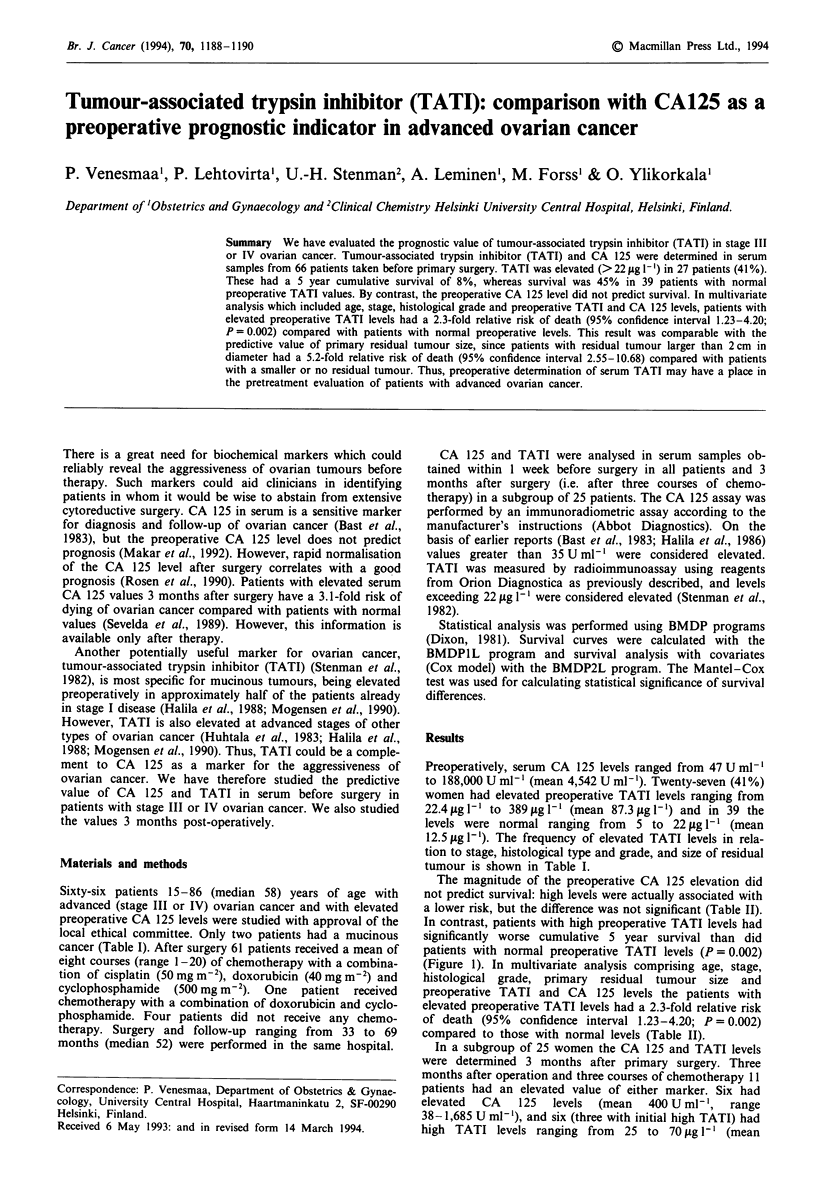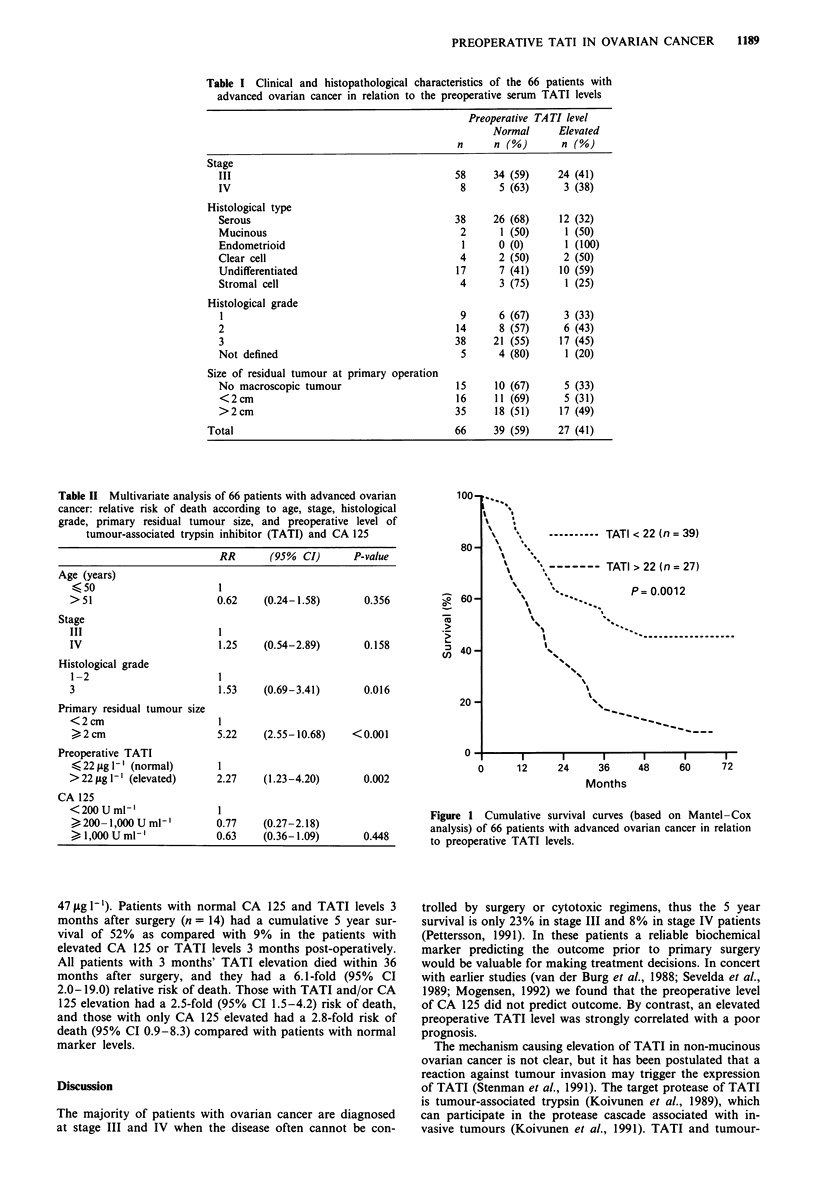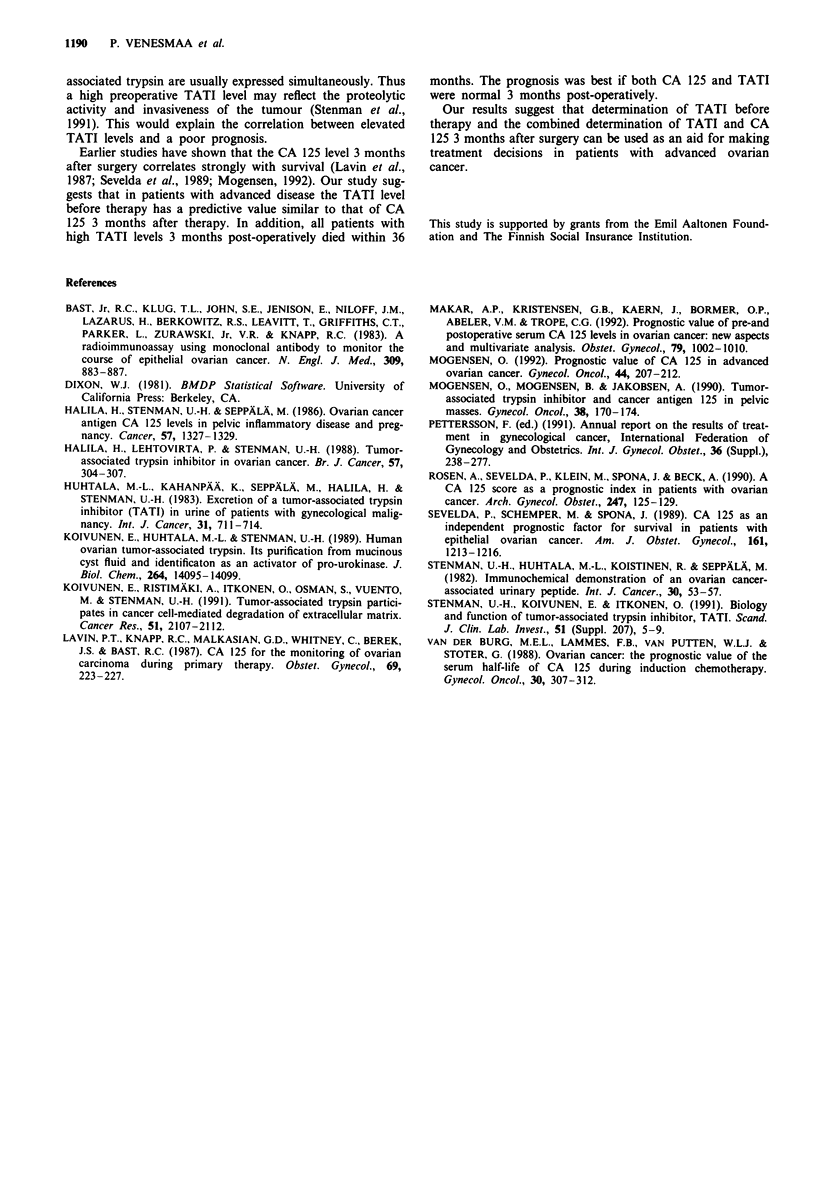# Tumour-associated trypsin inhibitor (TATI): comparison with CA125 as a preoperative prognostic indicator in advanced ovarian cancer.

**DOI:** 10.1038/bjc.1994.471

**Published:** 1994-12

**Authors:** P. Venesmaa, P. Lehtovirta, U. H. Stenman, A. Leminen, M. Forss, O. Ylikorkala

**Affiliations:** Department of Obstetrics and Gynaecology, Helsinki University Central Hospital, Finland.

## Abstract

We have evaluated the prognostic value of tumour-associated trypsin inhibitor (TATI) in stage III or IV ovarian cancer. Tumour-associated trypsin inhibitor (TATI) and CA 125 were determined in serum samples from 66 patients taken before primary surgery. TATI was elevated (> 22 micrograms l-1) in 27 patients (41%). These had a 5 year cumulative survival of 8%, whereas survival was 45% in 39 patients with normal preoperative TATI values. By contrast, the preoperative CA 125 level did not predict survival. In multivariate analysis which included age, stage, histological grade and preoperative TATI and CA 125 levels, patients with elevated preoperative TATI levels had a 2.3-fold relative risk of death (95% confidence interval 1.23-4.20; P = 0.002) compared with patients with normal preoperative levels. This result was comparable with the predictive value of primary residual tumour size, since patients with residual tumour larger than 2 cm in diameter had a 5.2-fold relative risk of death (95% confidence interval 2.55-10.68) compared with patients with a smaller or no residual tumour. Thus, preoperative determination of serum TATI may have a place in the pretreatment evaluation of patients with advanced ovarian cancer.


Br. J. Cancer (1994), 70, 1188–1190 © Macmillan Press Ltd., 1994

### Tumour-associated trypsin inhibitor (TATI): comparison with CA125 as a preoperative prognostic indicator in advanced ovarian cancer

P. Venesmaa[1], P. Lehtovirta[1], U.-H. Stenman[2], A. Leminen[1], M. Forss[1] & O. Ylikorkala[1]

*Department of [1]Obstetrics and Gynaecology and [2]Clinical Chemistry Helsinki University Central Hospital, Helsinki, Finland.*

**Summary** We have evaluated the prognostic value of tumour-associated trypsin inhibitor (TATI) in stage III or IV ovarian cancer. Tumour-associated trypsin inhibitor (TATI) and CA 125 were determined in serum samples from 66 patients taken before primary surgery. TATI was elevated (>22 $\mu g\,l^{-1}$) in 27 patients (41%). These had a 5 year cumulative survival of 8%, whereas survival was 45% in 39 patients with normal preoperative TATI values. By contrast, the preoperative CA 125 level did not predict survival. In multivariate analysis which included age, stage, histological grade and preoperative TATI and CA 125 levels, patients with elevated preoperative TATI levels had a 2.3-fold relative risk of death (95% confidence interval 1.23–4.20; $P = 0.002$) compared with patients with normal preoperative levels. This result was comparable with the predictive value of primary residual tumour size, since patients with residual tumour larger than 2 cm in diameter had a 5.2-fold relative risk of death (95% confidence interval 2.55–10.68) compared with patients with a smaller or no residual tumour. Thus, preoperative determination of serum TATI may have a place in the pretreatment evaluation of patients with advanced ovarian cancer.

There is a great need for biochemical markers which could reliably reveal the aggressiveness of ovarian tumours before therapy. Such markers could aid clinicians in identifying patients in whom it would be wise to abstain from extensive cytoreductive surgery. CA 125 in serum is a sensitive marker for diagnosis and follow-up of ovarian cancer (Bast *et al.*, 1983), but the preoperative CA 125 level does not predict prognosis (Makar *et al.*, 1992). However, rapid normalisation of the CA 125 level after surgery correlates with a good prognosis (Rosen *et al.*, 1990). Patients with elevated serum CA 125 values 3 months after surgery have a 3.1-fold risk of dying of ovarian cancer compared with patients with normal values (Sevelda *et al.*, 1989). However, this information is available only after therapy.

Another potentially useful marker for ovarian cancer, tumour-associated trypsin inhibitor (TATI) (Stenman *et al.*, 1982), is most specific for mucinous tumours, being elevated preoperatively in approximately half of the patients already in stage I disease (Halila *et al.*, 1988; Mogensen *et al.*, 1990). However, TATI is also elevated at advanced stages of other types of ovarian cancer (Huhtala *et al.*, 1983; Halila *et al.*, 1988; Mogensen *et al.*, 1990). Thus, TATI could be a complement to CA 125 as a marker for the aggressiveness of ovarian cancer. We have therefore studied the predictive value of CA 125 and TATI in serum before surgery in patients with stage III or IV ovarian cancer. We also studied the values 3 months post-operatively.

### Materials and methods

Sixty-six patients 15–86 (median 58) years of age with advanced (stage III or IV) ovarian cancer and with elevated preoperative CA 125 levels were studied with approval of the local ethical committee. Only two patients had a mucinous cancer (Table I). After surgery 61 patients received a mean of eight courses (range 1–20) of chemotherapy with a combination of cisplatin (50 mg $m^{-2}$), doxorubicin (40 mg $m^{-2}$) and cyclophosphamide (500 mg $m^{-2}$). One patient received chemotherapy with a combination of doxorubicin and cyclophosphamide. Four patients did not receive any chemotherapy. Surgery and follow-up ranging from 33 to 69 months (median 52) were performed in the same hospital.

CA 125 and TATI were analysed in serum samples obtained within 1 week before surgery in all patients and 3 months after surgery (i.e. after three courses of chemotherapy) in a subgroup of 25 patients. The CA 125 assay was performed by an immunoradiometric assay according to the manufacturer's instructions (Abbot Diagnostics). On the basis of earlier reports (Bast *et al.*, 1983; Halila *et al.*, 1986) values greater than 35 U $ml^{-1}$ were considered elevated. TATI was measured by radioimmunoassay using reagents from Orion Diagnostica as previously described, and levels exceeding 22 $\mu g\,l^{-1}$ were considered elevated (Stenman *et al.*, 1982).

Statistical analysis was performed using BMDP programs (Dixon, 1981). Survival curves were calculated with the BMDP1L program and survival analysis with covariates (Cox model) with the BMDP2L program. The Mantel–Cox test was used for calculating statistical significance of survival differences.

### Results

Preoperatively, serum CA 125 levels ranged from 47 U $ml^{-1}$ to 188,000 U $ml^{-1}$ (mean 4,542 U $ml^{-1}$). Twenty-seven (41%) women had elevated preoperative TATI levels ranging from 22.4 $\mu g\,l^{-1}$ to 389 $\mu g\,l^{-1}$ (mean 87.3 $\mu g\,l^{-1}$) and in 39 the levels were normal ranging from 5 to 22 $\mu g\,l^{-1}$ (mean 12.5 $\mu g\,l^{-1}$). The frequency of elevated TATI levels in relation to stage, histological type and grade, and size of residual tumour is shown in Table I.

The magnitude of the preoperative CA 125 elevation did not predict survival: high levels were actually associated with a lower risk, but the difference was not significant (Table II). In contrast, patients with high preoperative TATI levels had significantly worse cumulative 5 year survival than did patients with normal preoperative TATI levels ($P = 0.002$) (Figure 1). In multivariate analysis comprising age, stage, histological grade, primary residual tumour size and preoperative TATI and CA 125 levels the patients with elevated preoperative TATI levels had a 2.3-fold relative risk of death (95% confidence interval 1.23–4.20; $P = 0.002$) compared to those with normal levels (Table II).

In a subgroup of 25 women the CA 125 and TATI levels were determined 3 months after primary surgery. Three months after operation and three courses of chemotherapy 11 patients had an elevated value of either marker. Six had elevated CA 125 levels (mean 400 U $ml^{-1}$, range 38–1,685 U $ml^{-1}$), and six (three with initial high TATI) had high TATI levels ranging from 25 to 70 $\mu g\,l^{-1}$ (mean

Correspondence: P. Venesmaa, Department of Obstetrics & Gynaecology, University Central Hospital, Haartmaninkatu 2, SF-00290 Helsinki, Finland.
Received 6 May 1993: and in revised form 14 March 1994.

**Table I** Clinical and histopathological characteristics of the 66 patients with advanced ovarian cancer in relation to the preoperative serum TATI levels

| | | *Preoperative TATI level* | |
|---|---|---|---|
| | *n* | *Normal n (%)* | *Elevated n (%)* |
| Stage | | | |
| III | 58 | 34 (59) | 24 (41) |
| IV | 8 | 5 (63) | 3 (38) |
| Histological type | | | |
| Serous | 38 | 26 (68) | 12 (32) |
| Mucinous | 2 | 1 (50) | 1 (50) |
| Endometrioid | 1 | 0 (0) | 1 (100) |
| Clear cell | 4 | 2 (50) | 2 (50) |
| Undifferentiated | 17 | 7 (41) | 10 (59) |
| Stromal cell | 4 | 3 (75) | 1 (25) |
| Histological grade | | | |
| 1 | 9 | 6 (67) | 3 (33) |
| 2 | 14 | 8 (57) | 6 (43) |
| 3 | 38 | 21 (55) | 17 (45) |
| Not defined | 5 | 4 (80) | 1 (20) |
| Size of residual tumour at primary operation | | | |
| No macroscopic tumour | 15 | 10 (67) | 5 (33) |
| <2 cm | 16 | 11 (69) | 5 (31) |
| >2 cm | 35 | 18 (51) | 17 (49) |
| Total | 66 | 39 (59) | 27 (41) |

**Table II** Multivariate analysis of 66 patients with advanced ovarian cancer: relative risk of death according to age, stage, histological grade, primary residual tumour size, and preoperative level of tumour-associated trypsin inhibitor (TATI) and CA 125

| | *RR* | *(95% CI)* | *P-value* |
|---|---|---|---|
| Age (years) | | | |
| ≤50 | 1 | | |
| >51 | 0.62 | (0.24–1.58) | 0.356 |
| Stage | | | |
| III | 1 | | |
| IV | 1.25 | (0.54–2.89) | 0.158 |
| Histological grade | | | |
| 1–2 | 1 | | |
| 3 | 1.53 | (0.69–3.41) | 0.016 |
| Primary residual tumour size | | | |
| <2 cm | 1 | | |
| ≥2 cm | 5.22 | (2.55–10.68) | <0.001 |
| Preoperative TATI | | | |
| ≤22 μg $l^{-1}$ (normal) | 1 | | |
| >22 μg $l^{-1}$ (elevated) | 2.27 | (1.23–4.20) | 0.002 |
| CA 125 | | | |
| <200 U $ml^{-1}$ | 1 | | |
| ≥200–1,000 U $ml^{-1}$ | 0.77 | (0.27–2.18) | |
| ≥1,000 U $ml^{-1}$ | 0.63 | (0.36–1.09) | 0.448 |

**Figure 1** Cumulative survival curves (based on Mantel–Cox analysis) of 66 patients with advanced ovarian cancer in relation to preoperative TATI levels.

47 μg $l^{-1}$). Patients with normal CA 125 and TATI levels 3 months after surgery ($n = 14$) had a cumulative 5 year survival of 52% as compared with 9% in the patients with elevated CA 125 or TATI levels 3 months post-operatively. All patients with 3 months' TATI elevation died within 36 months after surgery, and they had a 6.1-fold (95% CI 2.0–19.0) relative risk of death. Those with TATI and/or CA 125 elevation had a 2.5-fold (95% CI 1.5–4.2) risk of death, and those with only CA 125 elevated had a 2.8-fold risk of death (95% CI 0.9–8.3) compared with patients with normal marker levels.

### Discussion

The majority of patients with ovarian cancer are diagnosed at stage III and IV when the disease often cannot be controlled by surgery or cytotoxic regimens, thus the 5 year survival is only 23% in stage III and 8% in stage IV patients (Pettersson, 1991). In these patients a reliable biochemical marker predicting the outcome prior to primary surgery would be valuable for making treatment decisions. In concert with earlier studies (van der Burg *et al.*, 1988; Sevelda *et al.*, 1989; Mogensen, 1992) we found that the preoperative level of CA 125 did not predict outcome. By contrast, an elevated preoperative TATI level was strongly correlated with a poor prognosis.

The mechanism causing elevation of TATI in non-mucinous ovarian cancer is not clear, but it has been postulated that a reaction against tumour invasion may trigger the expression of TATI (Stenman *et al.*, 1991). The target protease of TATI is tumour-associated trypsin (Koivunen *et al.*, 1989), which can participate in the protease cascade associated with invasive tumours (Koivunen *et al.*, 1991). TATI and tumourassociated trypsin are usually expressed simultaneously. Thus a high preoperative TATI level may reflect the proteolytic activity and invasiveness of the tumour (Stenman et al., 1991). This would explain the correlation between elevated TATI levels and a poor prognosis.

Earlier studies have shown that the CA 125 level 3 months after surgery correlates strongly with survival (Lavin et al., 1987; Sevelda et al., 1989; Mogensen, 1992). Our study suggests that in patients with advanced disease the TATI level before therapy has a predictive value similar to that of CA 125 3 months after therapy. In addition, all patients with high TATI levels 3 months post-operatively died within 36 months. The prognosis was best if both CA 125 and TATI were normal 3 months post-operatively.

Our results suggest that determination of TATI before therapy and the combined determination of TATI and CA 125 3 months after surgery can be used as an aid for making treatment decisions in patients with advanced ovarian cancer.

This study is supported by grants from the Emil Aaltonen Foundation and The Finnish Social Insurance Institution.

### References

BAST, Jr, R.C., KLUG, T.L., JOHN, S.E., JENISON, E., NILOFF, J.M., LAZARUS, H., BERKOWITZ, R.S., LEAVITT, T., GRIFFITHS, C.T., PARKER, L., ZURAWSKI, Jr, V.R. & KNAPP, R.C. (1983). A radioimmunoassay using monoclonal antibody to monitor the course of epithelial ovarian cancer. *N. Engl. J. Med.*, **309**, 883–887.

DIXON, W.J. (1981). *BMDP Statistical Software*. University of California Press: Berkeley, CA.

HALILA, H., STENMAN, U.-H. & SEPPÄLÄ, M. (1986). Ovarian cancer antigen CA 125 levels in pelvic inflammatory disease and pregnancy. *Cancer*, **57**, 1327–1329.

HALILA, H., LEHTOVIRTA, P. & STENMAN, U.-H. (1988). Tumor-associated trypsin inhibitor in ovarian cancer. *Br. J. Cancer*, **57**, 304–307.

HUHTALA, M.-L., KAHANPÄÄ, K., SEPPÄLÄ, M., HALILA, H. & STENMAN, U.-H. (1983). Excretion of a tumor-associated trypsin inhibitor (TATI) in urine of patients with gynecological malignancy. *Int. J. Cancer*, **31**, 711–714.

KOIVUNEN, E., HUHTALA, M.-L. & STENMAN, U.-H. (1989). Human ovarian tumor-associated trypsin. Its purification from mucinous cyst fluid and identificaton as an activator of pro-urokinase. *J. Biol. Chem.*, **264**, 14095–14099.

KOIVUNEN, E., RISTIMÄKI, A., ITKONEN, O., OSMAN, S., VUENTO, M. & STENMAN, U.-H. (1991). Tumor-associated trypsin participates in cancer cell-mediated degradation of extracellular matrix. *Cancer Res.*, **51**, 2107–2112.

LAVIN, P.T., KNAPP, R.C., MALKASIAN, G.D., WHITNEY, C., BEREK, J.S. & BAST, R.C. (1987). CA 125 for the monitoring of ovarian carcinoma during primary therapy. *Obstet. Gynecol.*, **69**, 223–227.

MAKAR, A.P., KRISTENSEN, G.B., KAERN, J., BORMER, O.P., ABELER, V.M. & TROPE, C.G. (1992). Prognostic value of pre-and postoperative serum CA 125 levels in ovarian cancer: new aspects and multivariate analysis. *Obstet. Gynecol.*, **79**, 1002–1010.

MOGENSEN, O. (1992). Prognostic value of CA 125 in advanced ovarian cancer. *Gynecol. Oncol.*, **44**, 207–212.

MOGENSEN, O., MOGENSEN, B. & JAKOBSEN, A. (1990). Tumor-associated trypsin inhibitor and cancer antigen 125 in pelvic masses. *Gynecol. Oncol.*, **38**, 170–174.

PETTERSSON, F. (ed.) (1991). Annual report on the results of treatment in gynecological cancer, International Federation of Gynecology and Obstetrics. *Int. J. Gynecol. Obstet.*, **36** (Suppl.), 238–277.

ROSEN, A., SEVELDA, P., KLEIN, M., SPONA, J. & BECK, A. (1990). A CA 125 score as a prognostic index in patients with ovarian cancer. *Arch. Gynecol. Obstet.*, **247**, 125–129.

SEVELDA, P., SCHEMPER, M. & SPONA, J. (1989). CA 125 as an independent prognostic factor for survival in patients with epithelial ovarian cancer. *Am. J. Obstet. Gynecol.*, **161**, 1213–1216.

STENMAN, U.-H., HUHTALA, M.-L., KOISTINEN, R. & SEPPÄLÄ, M. (1982). Immunochemical demonstration of an ovarian cancer-associated urinary peptide. *Int. J. Cancer.*, **30**, 53–57.

STENMAN, U.-H., KOIVUNEN, E. & ITKONEN, O. (1991). Biology and function of tumor-associated trypsin inhibitor, TATI. *Scand. J. Clin. Lab. Invest.*, **51** (Suppl. 207), 5–9.

VAN DER BURG, M.E.L., LAMMES, F.B., VAN PUTTEN, W.L.J. & STOTER, G. (1988). Ovarian cancer: the prognostic value of the serum half-life of CA 125 during induction chemotherapy. *Gynecol. Oncol.*, **30**, 307–312.